# Determinants of allocative and economic efficiency in crop-livestock integration in western part of Ethiopia evidence from Horro district: data envelopment approach

**DOI:** 10.1016/j.heliyon.2021.e07390

**Published:** 2021-06-24

**Authors:** Tolesa Tesema

**Affiliations:** Department of Agricultural Economics, Wollega University, P.O. Box 395, Ethiopia

**Keywords:** Efficiency analysis, Tobit, Non-parametric methods and data envelopment analysis

## Abstract

Sustainable development can be achieved through integrations of livestock and crops farm levels that generate higher economic efficiency in saving production costs through combining of crop and livestock. So, this study analyzed the allocative and economic efficiency of smallholder farmers in mixed crop-livestock production in Ethiopia evidence from Horo district using cross sectional data collected from 152 households in 2019/2020 cropping season by using structured questionnaires’. Data envelopment analysis was used to estimate the allocative and economic efficiency score. From the results of data envelopment analysis mean allocative efficiency was (57.0%) and economic efficiency (38.4%). Thus existence of about 61.6% economic inefficiency in the production of mixed crop-livestock production. Moreover, Tobit regression model results show that allocative efficiency affected by extension, off-non-farm and education levels of household positively. While economic efficiency positively affected by credit use, terrace and extension service positively and distance to the market negatively. Hence government should take steps for the improvement in education levels of household, development of terrace, expansion of nonfarm sectors and reform with extension service.

## 1 Introduction

Agriculture is primary economic sector for decreasing poverty and advancing realization of food security in Sub-Saharan Africa (World Bank, 2019). Likewise it also plays critical role in Ethiopia's development, providing the output required to feed a growing population, in the industrialization and overall transformation of the broader economy that accounts about 40% of the country's gross domestic product and nearly 70% of its employment (Agricultural Transformation Agency, 2015). In spite of this contribution agricultural productivity is lows, the high dependency on traditional, rain-fed farming in small and fragmented landholdings (African Development Bank, 2016). Thus in orders to improve low agricultural productivity the government of Ethiopia has made real efforts to improve smallholder performance through agricultural extension service programs mainly focused on input supply via credit systems and training for improved crop management recognizing the low productivity of agriculture and the potential contribution of smallholder agriculture to national economic growth and food security (Berhanu, 2009). Hence the effectiveness, worthiness and yield improvement schemes in the agricultural sector facilitated through proper advance and distribution of available technologies, implementing extension system according to the direction stated and undertaking the challenges which constrained the achievement of potential production capacity and improving the efficiency of the sector (Federal democratic republic of Ethiopia, 2016). In addition improving efficiency performance may need actions at the farm, policy and academic levels (Bouali, 2013). The higher the agriculture's supplies of resources the higher the efficiency mixed farming type of agricultural production (Marta and Katarzyna, 2020). Some of the studies such as (Ogunniyi, 2015; Jema, 2008; Abdulai et al., 2013) undertake the study on efficiency analysis. But this study estimate efficiency of resource use in mixed crop-livestock producers by adding more factors such as construction of terrace and integration of off-non-farm activities and considering the integration crop livestock from diversified farming by appling data envelopment approach than only specialized farming household.

## 2 Methodology

Data envelopment analysis were used in this study which is both non-parametric and non-stochastic since it does not impose any a priori parametric restrictions on the underlying frontier technology (because it does not cause any functional form to be specified) and doesn't entail any distributional assumption for the technical inefficiency term (Fare et al., 1985). Charnes et al. (1978) proposed a model which had an input-oriented constant return to scale model of data envelopment analysis. When input prices are available, one runs the constant return to scale cost minimization data envelopment analysis model (Fare et al., 1985) as:

(1) $$\begin{array}{l} \min{\;}_{\lambda xi*}{w^{\prime }}_{i}x_{i}^{*}\\ subject\;to-y_{i}+Y\lambda \geq 0\\ x_{i}^{*}-x\lambda \geq 0\\ \;\lambda \geq 0 \end{array}$$

where, wi = vector of input prices for the samples farmers such rental values of land per hectare 3000, mean price of labor were 40 birr, average price of oxen were 65 birr and price of each material inputs were material inputs collected and their average price were 250 birr such as (seed, feed, forage, urea, fertilizers and chemicals) , x∗ i is the cost minimizing vector of input quantities for the sample farmers such as amount of land in hectare, labor in man-day, oxen in oxen day and material input in birr given the average input prices, ; Y stands for sxn output matrix representing data for all n farmers and yi is output levels such as output of crop in Ethiopian birr and livestock in Ethiopian birr was entered in data envelopment analysis. λ is an nx1 vector of constants.

The cost efficiency or economic efficiency measures then estimated as the ratio of minimum cost to the observed cost. Thus:

(2) EECRS=wi′xi∗wi′xi

where EE_CRS_ is economic efficiency under constant return to scale, wi = vector of input prices and xi is cost minimizing input.

The allocative efficiency index can then be calculated residual from the technical efficiency measure. Economic efficiency index estimated in equation as:

(3) $$AE_{CRS}=\frac{EE_{CRS}}{TE_{CRS}}=\frac{w_{i}^{\prime }x_{i}^{*}}{w_{i}^{\prime }TE_{CRS}}$$

where AA_CRS_ is economic efficiency under constant return to scale, EECRS is economic efficiency under constant return to scale and TE_CRS_ technical efficiency under constant return to scale, wi is vector of input price and xi is cost minimizing input (Farrell, 1957).

To estimate factors that affect efficiency differentials in the study area the researchers used the Tobit models following Gujarati (2004). Tobit regression is specified as:

(4) $$y_{i}^{*}=\delta o+\delta_{m}Z_{im}+\mu$$

where $y_{i}*-$ Latent variable representing the technical, allocative and economic efficiency scores of sample household.

$z_{im}-$ The number of factors affecting efficiency such as credit use, livestock ownership in tropical livestock unit, age of household head in year, terrace contraction, sex of the household head, distance to nearest markets in minute, participation off/non-farm activities integration, extension contact in frequency of contacts, family size in adult equivalent and education levels in year of schooling
δ− A vector of parameter to be estimated
μ− Error term that is independently and normally distributed with zero mean and variance $\delta^{2}$

Denoting $y_{i}$ as observed variables,

(5) $$y_{i}=\left\{ \begin{array}{l} 1\;if\;y_{i}^{*}\geq 1\\ y_{i}^{*}\;if\;0<y_{i}^{*}<1\\ 0\;if\;y_{i}^{*}\leq 0\; \end{array}\right.$$

By following McDonald and Moffitt (1980), Greene (1980) from the likelihood function decomposition of marginal effects was proposed as follows two-limit Tobit model (Emana et al., 2012a):

The unconditional expected value of the dependent variable

(6) $$\frac{\partial E\left(y\right)}{\partial X_{j}}=\left[\phi \left(Z_{u}\right)-\phi \left(Z_{L}\right)\right].\frac{\partial E\left(y^{*}\right)}{\partial X_{j}}+\frac{\partial \left[\phi \left(Z_{u}\right)-\phi \left(Z_{L}\right)\right]}{\partial X_{j}}+\frac{\partial \left[1-\phi \left(Z_{u}\right)\right]}{\partial X_{j}}$$

The expected value of the dependent variable conditional upon being between the limits

(7) $$\frac{\partial E\left(Y^{*}\right)}{\partial X_{j}}=\beta m.\left[1+\frac{\left\{Z_{L}\varphi \left(Z_{L}\right)-Z_{u}\varphi \left(Z_{u}\right)\right\}}{\left\{\phi \left(Z_{u}\right)-\phi \left(Z_{L}\right)\right\}}\right]-\left[\frac{{\left\{\varphi \left(Z_{L}\right)-\varphi \left(Z_{u}\right)\right\}}^{2}}{{\left\{\phi \left(Z_{u}\right)-\phi \left(Z_{L}\right)\right\}}^{2}}\right]$$

The probability of being between the limits

(8) $$\frac{\partial \left[\phi \left(Z_{u}\right)-\phi \left(Z_{L}\right)\right]}{\partial X_{j}}=\frac{\beta_{m}}{\sigma }\left[\varphi \left(Z_{L}\right)-\varphi \left(Z_{u}\right)\right]$$

where = the cumulative normal distribution,

∅ = the normal density function
ZL = $-\frac{X_{i}^{\prime }\beta }{\sigma }$ and Zu = $\frac{\left(1-X_{i}^{\prime }\beta \right)}{1}$ are standardized variables that came from the likelihood function given the limits of y∗ and σ = standard deviation of the model.

## 3 Data and sampling procedures

### 3.1 Study area

**Figure 1:**
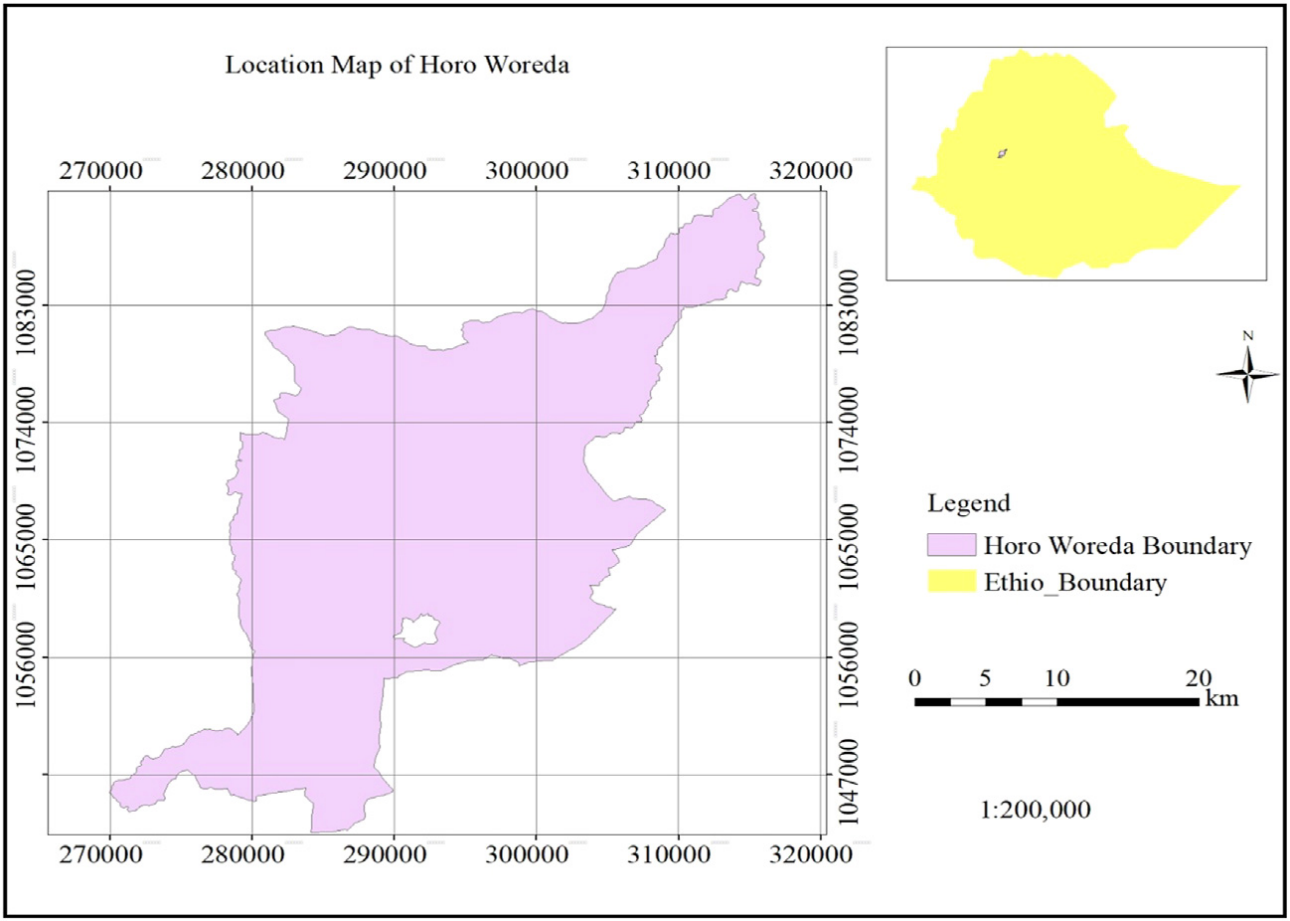
Map of the study area. Source: Geographical information system output, 2020.

Data used in this study was collected from Horro district which is located in Horro Guduru Wollega Zone which is one of the zone of oromia national regional state. The mean annual rain fall of the district is 1566 mm. The mean temperature is about 16.6 °C and the minimum temperature is 10.78^0^ were as the mean maximum temperature is 22.32 °C.Agro ecology of the district is dega (43%), woina dega (55.56%) and Kola (1.24) (Figure 1). The district is bordered by Jarte Jardaga district in north, Jimma ganati district in south East and Abe Dongoro district in north and Abay-coman distinct in east. The district has the total land area of 96,638.8km^2^. Mixed farming system is the main production activities in the study area (Horro Guduru office of agriculture and natural resource, 2019). Thus this study considers all crop and livestock producer households during production year.

### 3.2 Output-input variables data

In the first part of the data collections the value of crop and livestock output was obtained from output of crop such as wheat, barley, teff, maize, niger seed, potato, pea and bean was collected. From the livestock poultry and milk produced was collected from sample farmers. This aggregate output of crops and livestock that the sample household produced during the production period 2019/2020 was multiplied by their market price were to obtain the value of output of crop and livestock of the farm. The monthly market prices were collected from Horro district department of agriculture and rural development office. Then in these study values of crop and livestock were considered as dependent variables to production efficiency of sample household and independent variables were the amount of input available had positive impact on the output in the production such as land; labor oxen and material inputs are collected.

### 3.3 Data, data collection techniques and sampling procedures

The authors used primary data collected from 152 households with information collected at household levels by using structured questionnaires’. The data collected include plot level output of crop-livestock such as wheat, maize, teff barley, Niger seed, potato, faba been and pea and the inputs used in the production process such as size of land in hectare, amount of Labour in man day, the numbers of oxen used in oxen day, and material input used and the socioeconomic and farm-specific characteristics. During interview, the authors as well as respective enumerators provided enough evidence about the objectives of the study to avoid potential bias from the sample households in responding to questions. During data collection researchers used all ethical consideration from Wollega University committee. In this study two stage sampling techniques were used to select sample households. In the first stage 3 Kebeles namely Didibe Kistana, Loti Ano and Gitilo Najo were randomly selected from 11 Kebeles exit in the Horo district. In second stage from three Kebeles, 152 households were selected for survey based on Yemane formula at 8% levels of precision from 5703 total household (Yemane, 1967). Households that produces crop-livestock were selected by probability proportional to sample size. Accordingly 35 samples from Didibe Kistana, 90 sample from loti ano and 27 samples from Gitilo were selected from the district.

## 4 Empirical model

### 4.1 The descriptive statistics of output and input variables used in data envelopment methods

Output and input variables from crop and livestock were used in data envelopment analysis models to estimate the levels of allocative and economic efficiency of crop-livestock production. The value of crop and livestock outputs were derived from output of crop and livestock products that farmers produce in a given production year 2019/2020. These outputs were multiplied by their average market price to obtain the value of crop and livestock output. The average value of the output was 40,019.54 Ethiopian birr with minimum of 810 Ethiopian birr and maximum values of 97,800 Ethiopian birr. The average total land area (grazing and cultivated land) in the study area during a production year was 0.96 ha which ranged from 0.125 to 2.63 ha. This mean land holding is less than the result which is obtained by Teshome et al. (2021) that addressed 1.59 in south east Ethiopia. Controversy the result is higher than study made by Headey Dereje and Taffesse (2014). The mean of the oxen power and human labor that farm households used was 36.07 in oxen days and 203.57 in man-days and 28, consistently. The mean of the material inputs applied by the smallholder farmers was 5076.38 Ethiopia birr with a lowest of 892.5 Ethiopian birr and maximum of 13371.09 (Table 1).

**Table 1:** Descriptive results of input-output variables.

Variable	Mean	SD	Min	Max
Crop output in Ethiopian birr	40019.54	23932.04	810	97800
Livestock output in Ethiopian birr	6395.609	2665.138	0	14715
Land in hectare	0.96	0.51	0.125	2.63
Oxen in oxen day	36.07	18.2	4	98
Labor in man day	203.57	167.87	16.2	1122.2
Material input in Ethiopian birr	5076.38	2298.34	892.5	13371.09

1 birr is the local currency which is exchanged about 41 birr for an American dollar.

Source: Descriptive model result.

The results showed that the mean levels of allocative and economic efficiency scores were 57% and 38.4% respectively. The mean level of economic efficiency indicates that farmers could reduce current average cost of production by 62.6% to achieve the potential minimum cost of production relative to the efficient farmers given the current output level. The data envelopment analysis result of allocative efficiency and economic efficiency scores confirmed that almost all farm households (96.71%) were less efficient or inefficient were as 3.39 achieved allocative and economic efficiency score of 1.0 (Table 2). These mean levels of efficiencies are comparable with the results from other similar studies in Ethiopia. For example, Mustefa et al. (2017) obtained mean allocative and economic efficiencies 37.45% and 30.62% which is below this finding. Controversy Beneberu et al. (2018) obtained mean allocative and economic efficiencies 69% and 53% which is above this finding. Also Alene and Rashid (2005) found the mean allocative and economic efficiencies of 83% and 56% for traditional maize producers and 77 and 61% respectively for hybrid maize producing farmers in Eastern Ethiopia which is above this finding. Lastly Hassen (2011) also found the mean allocative and economic efficiency that 70% and 40% which is above this finding in north eastern Ethiopia.

**Table 2:** Data envelopment analysis estimates.

	Allocative efficiency constant return to scale	Allocative efficiency variable return to scale	Economic efficiency constant return to scale	Economic efficiency variable return to scale
Mean	0.563	0.570	0.373	0.384
SD	0.135	0.147	0.177	0.197
Minimum	0.235	0.235	0.103	0.103
Maximum	1	1	1	1

Where SD = standard deviation.

Source: Data Envelopment model result.

### 4.2 Frequency distributions of allocative and economic efficiency of mixed crop-livestock production system

**Figure 2:**
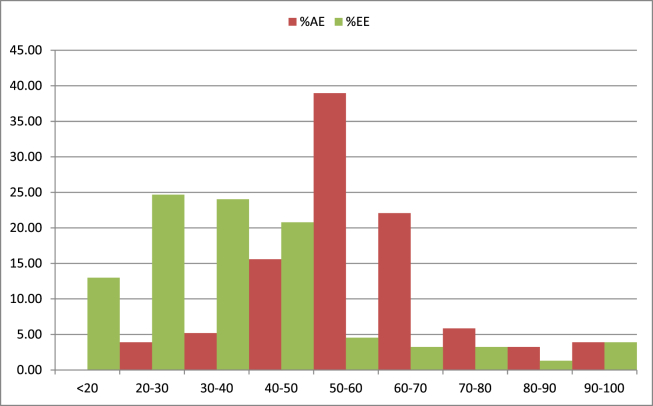
Distribution of allocative efficiency and economic efficiency score. Source: Descriptive model result.

From the survey results allocatively 24.67 percent of the farmers were half or less as efficient as the most efficient farmers. While 38.96 % of the farmers were seen to have allocative efficiency 50–60 percent. Regarding to economic efficiency about 83.55% of the entire households sampled was operating with an economic efficiency of not more than 0.5 (Figure 2). This show low efficiency score in the study area. This result is consistent with Joneydi (2012) which showed that inefficiency and low-productivity is major characteristics of developing country in mixed crop-livestock production.

### 4.3 Determinants of efficiency differential among farm household in mixed crop-livestock

After measuring levels of farmers' efficiency in mixed crop-livestock integration allocative and economic efficiency estimates derived from the data envelopment model were regressed on demographic, socioeconomic, farm and institutional variables that explain variations in efficiency across farm households using two limits Tobit regression model.

#### 4.3.1 Credit uses

The result also indicated that credit used had a positive sign and statistically significant effect on economic efficiency level at 5% level of significance. This advocates that on average households who use credit tend to unveil higher levels of efficiency. Moreover, a change in the dummy variable representing the uses credit by the household ordered from 0 to 1 would increase the probability of the farmers being economically efficient by about 5.49% and change the expected value of economic efficiency by about 4.62% with an overall increase in the probability and the level of efficiencies by 4.01%, respectively (Table 4). A significant positive influence was also reported by (Awudu and Wallace, 2016; Assefa et al., 2019).

#### 4.3.2 Off-non-farm integration of household head

The coefficient of participation in non-farm activity was positive and significant affects allocative efficiency at 5% significance level. Participation in non-farm activity affect efficiency positively for the reason that the income obtained from such activities could be used for the purchase of agricultural inputs and may be because of the availability of non-farm income shifts the cash constraint outwards and enables farmers to make timely purchase of those inputs which they cannot offers from on farm income. In addition, the computed marginal effect revealed, a change in a dummy variable participation in non-farm integration would increase the probability of the farmer being allocatively efficient by about 0.53% and the expected value of allocative and economic efficiencies by about 4.25 % with an overall increase in the probability and levels of allocative efficiencies by 4.43% (Table 3). Awudu and Wallace (2016), Bekele (2013) also reported similar results.

**Table 3:** Coefficient and marginal effect of determinants of allocative efficiency.

Variables	Allocative efficiency		Marginal effect		
	Coefficient	Standard error	$\frac{\partial E\left(y\right)}{\partial X_{j}}$	$\frac{\partial E\left(y^{*}\right)}{\partial X_{j}}$	$\frac{\partial \left(\varphi \left(Z_{U}-Z_{L}\right)\right)}{\partial X_{j}}$
Credit	0.01831266	0.0222487	0.0181993	0.0174586	0.0021727
Livestock holding	-0.00295842	0.001966	-0.002941	-0.0028251	-0.0003302
Age	0.00021798	0.0009876	0.0002167	0.0002082	0.0000243
Terrace	0.00770554	0.0216726	0.0076598	0.0073559	0.0008713
Sex	0.00561222	0.028389	0.0055781	0.0053533	0.0006541
Distance to market	-0.00051842	0.0007583	-0.0005154	-0.0004951	-0.0000579
Off-non farm integration	0.04464836∗∗	0.0223169	0.0443668	0.0425487	0.0053245
Extension service	0.00263295∗∗	0.0011745	0.0026175	0.0025143	0.0002939
Family size	0.00024872	0.0077346	0.0002473	0.0002375	0.0000278
Education levels	0.00792671∗∗∗	0.0029144	0.0078801	0.0075696	0.0008848
_cons	.8327634∗∗∗	0.0700458			

Note: ∗, ∗∗ and ∗∗∗ refers to level of significance at 10, 5 and 1% respectively.

$\frac{\partial E\left(y\right)}{\partial X_{j}}$, $\frac{\partial E\left(y^{*}\right)}{\partial X_{j}}$ and $\frac{\partial \left(\varphi \left(Z_{U}-Z_{L}\right)\right)}{\partial X_{j}}$

Show total changes, expected changes and changes of probability in levels of allocative and economic efficiency changes due to determinant factors.

Source: Tobit model result presented using table 3.

#### 4.3.3 Contractions of terrace

Economic efficiency was influenced by contraction of terrace on farm land at 5% levels of significance positively. This is due the reason that the contractions of terrace by the community increase the fertility of the land that in turns increase the efficiency of mixed crop livestock production of smallholder's farmers. More over grass can growth on farm land that is used as source of feed for livestock. Likewise, the marginal effect from the Tobit model indicated, a change in a contractions of terrace from (participated to not participated) would increase the probability of a farmer being economically efficient by 3.72%, 4.49% and 5.31% respectively (Table 4). This study is consistent with Mesay et al. (2013); Geta et al. (2010) and Berhan (2015)

**Table 4:** Coefficient and marginal effect of determinants of economic efficiency.

Variables	Economic efficiency		Marginal effect		
	Coefficient	Standard error	$\frac{\partial E\left(y\right)}{\partial X_{j}}$	$\frac{\partial E\left(y^{*}\right)}{\partial X_{j}}$	$\frac{\partial \left(\varphi \left(Z_{U}-Z_{L}\right)\right)}{\partial X_{j}}$
Credit	0.05823401∗∗	0.0284316	0.0549012	0.0462092	0.0401808
Livestock holding	-0.00409357	0.0025109	-0.0038838	-0.0032985	-0.0025993
Age	-0.00074603	0.0012597	-0.0007078	-0.0006011	-0.0004737
Terrace	0.05619436∗∗	0.0276702	0.0531327	0.0449286	0.037294
Sex	-0.03104122	0.0362364	-0.0296308	-0.0254536	-0.0178472
Distance to market	-.00271606∗∗∗	0.0009677	-0.0025769	-0.0021885	-0.0017247
Off-non-farm integration	0.0352062	0.0285078	0.0333593	0.0282851	0.0227362
Extension service	0.00338301∗∗	0.001499	0.0032097	0.0027259	0.0021482
Family size	-0.00808309	0.0098724	-0.0076689	-0.0065131	-0.0051326
Education levels	0.0029469	0.0037193	0.0027959	0.0023745	0.0018712
_cons	0.44507156∗∗∗	0.089316	0.0549012	0.0462092	0.0401808

Note: ∗, ∗∗ and ∗∗∗ refers to level of significance at 10, 5 and 1% respectively.

$\frac{\partial E\left(y\right)}{\partial X_{j}}$, $\frac{\partial E\left(y^{*}\right)}{\partial X_{j}}$ and $\frac{\partial \left(\varphi \left(Z_{U}-Z_{L}\right)\right)}{\partial X_{j}}$

Show total changes, expected changes and changes of probability allocative and economic efficiency changes due to determinant factors.

Source: Tobit Model result.

#### 4.3.4 Distance to the market

The coefficient of distance to the market was statistically significant factors that affect economic efficiency at 1% levels of significance negatively. This is due to the reasons that to buy inputs for crop and livestock farmers in remote area can pay more cost of transportation. Additionally as distance from the market increase by 1 min the probability, mean and overall changes decrease by 0.17%,0.21% and 0.25% (Table 4). The similar result was obtained by Awudu and Wallace (2016); Moges (2018) and Tsegaye et al. (2019).

#### 4.3.5 Extension service

The positive coefficient of extension service which is significant affects both allocative and economic efficiency indicates that farmers who efficient in crop–livestock production tended to modify their production decisions to resonate with extension advice. Thus, they are able to add additional knowledge's crops and/or livestock to the initial commodity set to meet the maximum performance. The computed marginal effect indicated, a unit increase in the number of farm size would decrease the probability of a farmer being allocative and economic efficient by 0.029 and 0.21%, and the mean value of allocative and economic efficiency by about 0.25 and 0.27% with an overall increase in the probability and the level of allocative and economic efficiency by 0.26 and 0.32% (Tables 3 and 4). This result is similar with the findings of (Mustafa et al., 2017; Sisay et al., 2015 and Hunde and Abera, 2019).

#### 4.3.6 Educational levels of household heads

Education was statistically significant at 1% and affects positively the allocative efficiency of crop-livestock producers. This due to the reason that farmers can obtain knowledge in price determinations since that increases allocative efficiency of smallholder's farmers in mixed crop livestock production. Moreover as education increase by one year the probability of farmers being allocative efficient, mean efficiency and total change of allocative efficiency increase by 0.09, 0.75 and 0.78 percent (Table 3). Similar results were obtained in the works of (Moges, 2018; Sisay et al., 2015; Degefa et al., 2020) in Ethiopia.

## 5 Conclusion and policy implications

In the developing countries including Ethiopia improvement in efficiency of smallholder farmers has vital effect for the development approaches. Hence this study was investigating the determinants of allocative and economic efficiency of smallholder's farmers in crop-livestock integration in Horo district of Horo Guduru Wollega Zone oromia regional state, Ethiopia. In order to realize the stated objective the authors used primary data collected from 152 sample household using structured questionaries' and secondary data from district office, different journals and report. The mean allocative and economic efficiency obtained from data envelopment analysis variables return to scale were 57% and 38.4%.To find the factors that affect efficiency two limit Tobit regression model was used. According to the findings from the Tobit regression model off-non-farm integration, extension service and educational levels of household are found to be positively affecting allocative efficiency levels while uses of credit, contraction of terrace and extension service affect economic efficiency levels positively where distance to the market affect economic efficiency negatively. The policy implication drawn from this study was since coefficient of terrace of land is significant factors that affect the efficiency levels hence it is crucial to puts strategies for the development of different soil conservation mechanisms the increases fertility status of land in the study area. There should be policies and strategies toward expansion and promotion of off-non-farm sectors integration which offer rivets the excess labor from the agricultural sector and as additional income for farmer's livelihood improvements. These integrations can be achieved through participating market oriented production activities off-non-farm sectors such as hand craft, petty trade and resource extraction. Moreover, as the result indicates the extension contacts were positively affect efficiency of crop-livestock production. Thus government should develop network of crop extension agents and livestock extension agents to boost smallholder agriculture efficiency. Furthermore, uses of credit affect efficiency of smallholder's farmers positively. Thus governments and microfinance have to develop the micro finance institutions such as oromia credit and saving Share Company and wasasa micro finance institutions. Furthermore the available the credit service have to be expanded to the rural community at village levels since most of smallholder farmers pays the transport cost in addition to the interest that micro finance institutions get from credit collection. However, there is a need for further study of why farmers not repay accessed credit in the study area. Based on these finding educated farmers should inspire their knowledge and uneducated farmers have to acquire educations opportunity via adult education learning's and government in association with extension agents should educate the farmers on the vital input quantities to use on their farms during production process. These could enhance their efficiency and output levels which can lead to reduction in input wastage and so increase their income level and increase the gross domestic products of country in general.

## Declarations

### Author contribution statement

Tolesa Tesema: Conceived and designed the experiments; Performed the experiments; Analyzed and interpreted the data; Contributed reagents, materials, analysis tools or data; Wrote the paper.

### Funding statement

This work was supported by Wollega University.

### Data availability statement

No data was used for the research described in the article.

### Declaration of interests statement

The authors declare no conflict of interest.

### Additional information

No additional information is available for this paper.
